# A Comparison of Interpretable Machine Learning Approaches to Identify Outpatient Clinical Phenotypes Predictive of First Acute Myocardial Infarction

**DOI:** 10.3390/diagnostics14161741

**Published:** 2024-08-10

**Authors:** Matthew Hodgman, Cristian Minoccheri, Michael Mathis, Emily Wittrup, Kayvan Najarian

**Affiliations:** 1 Department of Computational Medicine and Bioinformatics, University of Michigan, Ann Arbor, MI 48109, USA; 2 Department of Anesthesiology, University of Michigan, Ann Arbor, MI 48109, USA; 3 Michigan Institute for Data Science, University of Michigan, Ann Arbor, MI 48109, USA; 4 Max Harry Weil Institute for Critical Care Research and Innovation, University of Michigan, Ann Arbor, MI 48109, USA; 5 Department of Emergency Medicine, University of Michigan, Ann Arbor, MI 48109, USA; 6 Department of Electrical Engineering and Computer Science, University of Michigan, Ann Arbor, MI 48109, USA

**Keywords:** artificial intelligence, acute myocardial infarction, computational phenotypes, interpretable machine learning

## Abstract

Background: Acute myocardial infarctions are deadly to patients and burdensome to healthcare systems. Most recorded infarctions are patients’ first, occur out of the hospital, and often are not accompanied by cardiac comorbidities. The clinical manifestations of the underlying pathophysiology leading to an infarction are not fully understood and little effort exists to use explainable machine learning to learn predictive clinical phenotypes before hospitalization is needed. Methods: We extracted outpatient electronic health record data for 2641 case and 5287 matched-control patients, all without pre-existing cardiac diagnoses, from the Michigan Medicine Health System. We compare six different interpretable, feature extraction approaches, including temporal computational phenotyping, and train seven interpretable machine learning models to predict the onset of first acute myocardial infarction within six months. Results: Using temporal computational phenotypes significantly improved the model performance compared to alternative approaches. The mean cross-validation test set performance exhibited area under the receiver operating characteristic curve values as high as 0.674. The most consistently predictive phenotypes of a future infarction include back pain, cardiometabolic syndrome, family history of cardiovascular diseases, and high blood pressure. Conclusions: Computational phenotyping of longitudinal health records can improve classifier performance and identify predictive clinical concepts. State-of-the-art interpretable machine learning approaches can augment acute myocardial infarction risk assessment and prioritize potential risk factors for further investigation and validation.

## 1. Introduction

An acute myocardial infarction (AMI), or “heart attack”, is myocardial necrosis due to sudden ischemia caused by blood clotting around ruptured or exposed plaque in the coronary arteries [1,2]. Globally, more than 3 million people have an AMI each year [3]. Fortunately, AMI incidence rates have declined as researchers and clinicians have identified and managed risk factors [4]. The vast majority of AMIs occur out of the hospital, where patients have limited monitoring [5]. Additionally, most reported AMIs are the patients’ first and are often unaccompanied by comorbidities [1,6]. These observations highlight the inherent difficulty in predicting AMI events.

There have been significant efforts to predict a variety of severe adverse cardiovascular events, including AMIs. Many studies predict AMI onset in patients while they are in the hospital [7,8,9,10]. However, ideally, prediction occurs earlier so clinicians can intervene to avoid hospitalization. Using electronic health record (EHR) data for over 20,000 AMI cases in a cohort of 2.27 million patients from the UCHealth hospital system, Mandair et al. predicted the 6 month risk of a first AMI using several machine learning models [11]. The best-performing model achieved an AUROC of 0.835 and F1 of 0.092. Unfortunately, their model exhibited poor calibration, ignored timing, and did not utilize laboratory values, nor did they provide any insight into how their model made predictions. Moore and Bell used XGBoost on data from over 500,000 patients in the UK Biobank to predict self-reported “heart attack” (11,849 cases) [12]. They interpreted their models using SHAP values. However, they did not give any information regarding the timing of recorded features or heart attack. Wang et al. predicted AMI within 10 years in 11,635 patients but did not provide any model interpretability [13]. Tsarapatsani et al. predicted 10 year AMI onset in a cohort of 3267 patients that had electrocardiogram and angiography data available, and used SHAP values for model explainability [14]. Sievering et al. predicted 5 year AMI onset in 500 patients with coronary artery disease using angiography images and 11 clinical features [15]. While significant effort has been put into predicting AMIs, the resulting models often focus on patients who already have cardiovascular comorbidities, ignore temporal relationships in the data, and lack sufficient interpretability and explainability.

Interpretability and explainability in machine learning models generally derive from two approaches: model structure and post hoc analyses. Common post hoc methods for quantifying feature importance include SHAP [16] and LIME [17] values, albeit with potentially questionable reliability [18]. Many canonical models incorporate inherent interpretability into their structure. Logistic regression models provide variable coefficients that indicate the effect of features on the outcome. Tree-based models like random forest [19] and Extreme Gradient Boosting (XGBoost) [20] compute feature importance based on location in trees and metrics like impurity and gain, respectively. Generalized additive models, such as the Explainable Boosting Machine (EBM) [21], learn a nonlinear function for each variable, or interaction of variables, which describes their impact on the model. Attention mechanisms embedded in deep learning models can explain what data heavily weighs the outcome and relationships between data. For example, TabNet provides global and local feature importance scores [22]. However, the interpretability and reliability of attention is disputed [23]. Matrix and tensor factorizations methods learn interpretable factors that provide a low-rank approximation of the data and can be used for clustering, phenotyping, dimensionality reduction, and feature engineering. Applied to EHR data, tensor factorization can automatically discover patterns of co-occurring medical variables across patients and their evolution across time [24]. This has proven useful as the irregular temporal nature of EHR data is a primary challenge. Fuzzy neural networks are models that use fuzzy logic within a neural network structure to map features to interpretable concepts and learn logical rules for prediction. Specifically, the tropical geometry fuzzy neural network (TGFNN) developed by Yao et al. has shown recent promise [25]. We employ several of these interpretable methods in this work.

In this work, we assess whether state-of-the-art interpretable machine learning approaches can learn clinical profiles that predict a patient’s first AMI, before hospitalization is required. We extract five years of longitudinal outpatient EHR data for patients without cardiovascular diagnoses before AMI onset, from the University of Michigan Health System (2698 positive and 5396 matched negative samples). Using tensor factorization, we reduce the dimensionality of the longitudinal health history while preserving interpretability and temporal relationships. Using the EHR phenotypes and other patient data, we train seven state-of-the-art interpretable machine learning models, including TGFNN, to predict AMI onset within six months. We evaluate whether incorporating temporal information via computational phenotyping improves model performance, overall model performance, and the consistency of important features. We present and clinically validate the learned phenotypes, rules, and relationships that explain the models’ predicted outcomes. We anticipate that these findings can assist researchers and clinicians in better understanding the risk factors of AMI, identifying at-risk patients, and providing preventative care.

## 2. Materials and Methods

### 2.1. Dataset

In this study, we used outpatient data collected from adult patients of the University of Michigan Health System (UMHS) from 1 January 2012 to 1 May 2023.

To define our cohort, we retrieved data from adult patients (23–89 years) who had at least three outpatient visits within the five years before their latest visit or their first cardiac diagnosis. We defined cardiac diagnoses as ICD9 codes 410.*–429.* and 785.0–785.1, and ICD10 codes I20.*–I52.* and R00.*. Cases, or positive samples, were defined as those patients in the cohort whose first cardiac diagnosis was an AMI (ICD9: 410.*; ICD10: I21.*). Controls, or negative samples, were any other UMHS patient who met the above criteria but did not have a cardiac diagnosis. We matched two negative patients to each positive patient based on sex, ±2 years in age (at time of AMI or last encounter), and ±2 points in the hospital frailty risk score [26]. We computed each patient’s hospital frailty risk score using all diagnoses on their EHR in the five-year period. Positive patients without control matches were excluded. This resulted in a cohort of 2641 positive patients (those who develop an AMI) and 5287 negative patients (those who do not develop an AMI). We split the patients into training and testing sets with a 70–30 split. For demographic information on the cohort, see Table 1.

### 2.2. Data Preprocessing

We extracted each patient’s data within the five years before their AMI or last recorded encounter. These data included time-dependent data like diagnoses, medications, vitals, laboratory results, and substance use. Time-independent data were also extracted, including demographics and family health history. We cleaned the data to remove erroneous and ambiguous values (e.g., text entry in numeric variable column, values outside of possible range, etc.). We converted all temperature values to Fahrenheit. Continuous variables like laboratory values and vitals were only included if >60% of patients in the training set had at least one measurement. We arbitrarily selected 60% as a missingness cutoff to prioritize the most common and accessible clinical variables as well as limit errors in downstream imputation. Removing rare variables reduces the need for data imputation and prioritizes results based off common, accessible variables. Categorical variables, like race, were one-hot encoded. We excluded patients missing information on their sex. We determined whether patients had a family history of cardiovascular diseases by whether they had at least one familial occurrence of heart disease, heart attack, coronary artery disease, heart failure, heart defect, aortic disease, sudden cardiac death, cardiomyopathy, cardiovascular disease, or rheumatic heart disease. We excluded procedure data. Diagnosis features were originally recorded as codes from the International Statistical Classification of Diseases (ICD) version 9 or 10 [27] and we converted all ICD9 codes to ICD10 via a conversion table provided at https://github.com/bhanratt/ICD9CMtoICD10CM, accessed on 3 March 2024. We removed all “Z” chapter ICD10 codes. Diagnoses were encoded as binary variables to indicate the presence of the ICD10 code, regardless of how often it was recorded. To condense the diagnosis data, we added the higher-level IDC10 categories as features if one of their children diagnoses was present, e.g., if a patient had ICD10 code E11.0 present, they would also have E11 marked as present. Medication information was also encoded as binary variables indicating its prescription at every encounter between its start and stop dates. Medication feature names were taken directly as recorded in the EHR. For both diagnosis and medication data, we employed carry forward imputation followed by zero imputation. We removed variables present in less than 1% of both case and control patients in the training set. All data preprocessing was completed in Python (Version 3.9) and all code used in the study is available at https://github.com/kayvanlabs/interpretable-ami-prediction, accessed on 3 March 2024.

We extracted three different sets of features from the data: (1) the demographics, or time-independent data, and latest recorded values within six months of AMI onset or the last encounter, (2) summary statistics of the entire five-year history, and (3) computational phenotypes of five-year health history using unsupervised tensor factorization. We selected these approaches for condensing patients EHR history because they are common, interpretable, easy to compute, and have been shown to be effective in other studies. Additionally, we evaluated the combination of the feature sets. In total, we tested six distinct feature sets:

- Latest data and demographics;
- Summary statistics;
- Computational phenotypes;
- Latest data, demographics, and summary statistics;
- Latest data, demographics, and computational phenotypes;
- Latest data, demographics, summary statistics, and computational phenotypes.

### 2.3. Latest Recorded Data of Health History

For each patient in the dataset, we extracted the most recent measurement of each variable before AMI onset or the last recorded visit, for positive and negative samples, respectively.

### 2.4. Summary Statistics of Health History

Summary statistics of clinical variables are fast to compute, easily understandable, and can be predictive of important outcomes [28]. We summarized laboratory and vital data over the five-year observation window by computing the mean, standard deviation, minimum, and maximum of each variable, for each patient. Categorical variables were aggregated by taking the maximum value, indicating whether a patient ever had the feature present in the five-year window. These statistics reduced the multiple, longitudinal measurements of each variable to a single, interpretable value.

### 2.5. Computational Phenotypes of Health History

Computational phenotyping of high-dimensional EHR data via tensor decomposition enables automated, low-dimensional representation of co-occurring medical events across patients, different types of variables, and time [24]. In this work, we use tensor decomposition to discover temporal, clinical phenotypes that act as interpretable features for downstream classification. While various tensor factorization approaches exist, we used the unsupervised, non-negative PARAFAC decomposition, with the hierarchical alternating least squares algorithm, implemented in TensorLy (Version 0.8.1) because of its simplicity, wide use, and ease of use [29]. Non-negative PARAFAC decomposition approximates the original data with the sum of rank-one component tensors. Each component tensor is defined by the outer product of vectors, one for each mode of the original data. The values in these vectors are learned via alternating least squares and describe the components. In this work, we decompose three-dimensional tensors with the modes: patients, time, and features. Thus, after applying non-negative PARAFAC decomposition, each component of the factorization can be interpreted as a phenotype defined by three vectors that encode the membership, weight, or importance of patients, time points, and features.

We learned temporal phenotypes for laboratory values and vitals separately from diagnoses and medication. All laboratory values and vitals for the five years preceding AMI onset or the patient’s last encounter were separated into ten, six-month segments. Only the last recorded value of each feature was kept per segment. To simplify computation and interpretation, each feature was discretized into quintiles based on the feature distributions in the training set. The resulting three-dimensional tensor representation of the data consisted of modes: patient × time × feature and size 7928 × 10 × 320.

We used diagnosis and medication data over the 5 observation years to generate temporal phenotypes. First, we split the 5 years into ten, six-month intervals. Within each interval, we recorded the diagnoses documented at encounters with a “1” and undocumented diagnoses as “0”. If there was no encounter in the six-month interval, diagnosis variables were left as null. We then performed carry forward imputation of all diagnosis codes until the next interval with an encounter. All remaining null values were imputed with zero. For medication data, we marked a “1” if the medication was prescribed during a given interval; otherwise a “0” was inserted. We formatted these data into a three-dimensional tensor of modes: patient × time × feature of size 7928 × 10 × 734.

**Figure A1:**
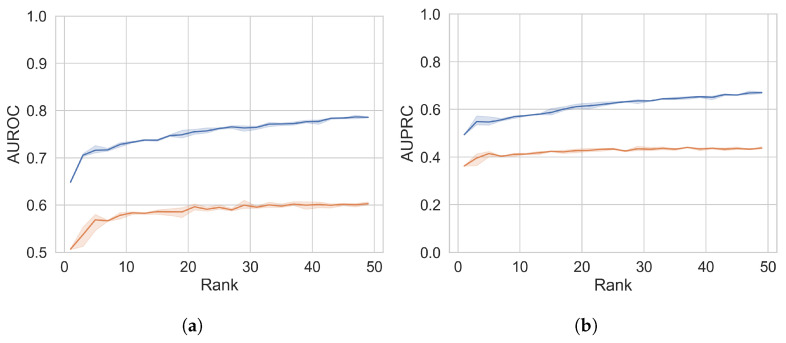

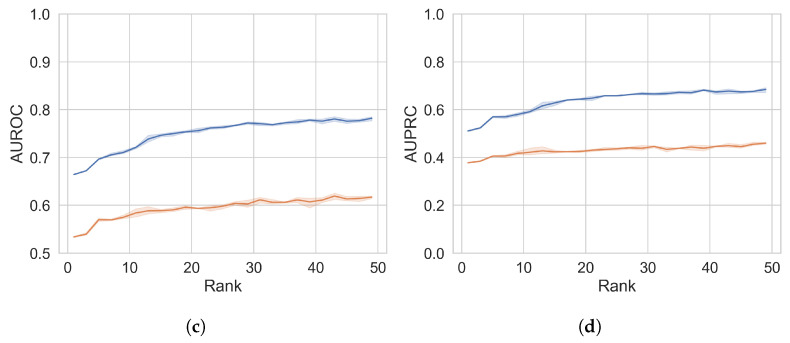
The predictive performance of random forest using features from different rank decompositions. The lines (blue = training set, orange = validation set) show the mean score of three replicates, with shaded regions showing 95% confidence intervals. (**a**) Mean AUROC of lab/vital phenotypes; (**b**) mean AUPRC of lab/vital phenotypes; (**c**) mean AUROC of Dx/Rx phenotypes; (**d**) mean AUPRC of Dx/Rx phenotypes.

Determining the optimal number of phenotypes, or rank, of a tensor decomposition is an open problem [24]. One common approach is to evaluate and plot the predictive performance of various ranks and choose the rank at the “elbow” of the curve—effectively identifying the rank after which performance increases are marginal. We carried this out by first factoring the training set using ranks at increments of two, between one and 50, running three replicates at each rank. Next, using the normalized patient membership to the phenotypes as features, we removed 30% of the training set for a validation set, trained a random forest model to discriminate between positive and negative samples, and visualized the performance according to common machine learning metrics, against the rank. For both the lab/vital and diagnoses/medications (Dx/Rx) phenotypes, the F1 score plateaued by a rank of ten. However, both AUROC and AUPRC gradually increased till approximately 30 for lab/vital phenotypes and continued to increase to a rank of 50 for Dx/Rx phenotypes (see Figure A1). We decided to use those ranks for each decomposition. While the predictive performance of the Dx/Rx phenotypes may continue to increase beyond rank 50, in practice, more phenotypes may become increasingly redundant or less interpretable.

Using the ranks of 30 and 50, we decomposed the training set tensors and extracted the learned lab/vital and Dx/Rx phenotypes, respectively. To determine the test set patients’ membership of these phenotypes, we fixed the feature and time dimensions of the phenotypes to those of the training set and then set the decomposition to fit only the patient membership mode. This projects the phenotypes onto the test set patients to determine their membership of each, without changing the phenotypes themselves. Lastly, we concatenated the lab/vital phenotype features with the Dx/Rx phenotype features into a single patient × feature table with 80 temporal phenotypes as features to describe each patient’s EHR history.

### 2.6. Feature Selection

We used the Minimum Redundancy and Maximum Relevance (mRMR) [30] approach to select the most relevant and least redundant subset of features from each feature set: latest/demographics, summary statistics, and computational phenotypes. We opted to use this feature selection approach because it not only identifies the most relevant features but also limits collinearity between the selected features, unlike other feature selection methods. Using mRMR can improve model performance, speed, and interpretability [31]. Feature relevance is determined by random forest feature importance and feature redundancy by Pearson’s correlation. To assess the optimal number of features for each feature set, we incrementally increased the number of features to use when running mRMR and then assessed their performance in three random forest models. By looking at the results, for each feature set, we determined a reasonably small number of features with near-optimal performance. We selected 20 features for the latest and demographic feature set, 30 for the summary statistics feature set, and 30 for the phenotypes feature set. After feature selection, if any variables had missing values, we imputed them using k-nearest neighbors, as implemented in Scikit-learn (Version 1.2.2) [32], fit on the training set only, and applied to both training and testing sets. Next, we performed the same experiment using combinations of the three feature sets and opted to use 20 features in the latest/demographic/summary statistics feature set, 30 in the latest/demographic/phenotypes feature set, and 60 in the “All” feature set (latest/demographic/summary statistics/phenotypes).

### 2.7. Model Training and Cross-Validation

We selected a set of machine learning models to evaluate in this work based on their interpretability and accessibility, including decision tree (DT), logistic regression with L2 penalty (LR), random forest (RF), EBM, XGBoost (XGB), TabNet (TNET), and TGFNN. We used the decision tree, logistic regression, and random forest implementations from scikit-learn (Version 1.2.2), the EBM implementation from InterpretML (Version 0.3.2) [16], and XGBoost (Version 1.7.5) [20], TabNet (Version 4.1.0) [22], and the TGFNN as described in [25] and implemented in Pytorch (Version 2.1.0). To compensate for dataset imbalance, we up-weighted the minority class (positive) and down-weighted the majority class (negative). In models not allowing class weights (EBM, XGBoost, and TabNet), we randomly downsampled the majority class (negative) to a 1:1 ratio with the minority class (positive).

We performed three-fold cross-validation on the 90% of the training set (10% withheld as a validation set) to determine the optimal hyperparameters for each model. We randomly sampled 500 combinations of hyperparameters for each model, except TGFNN. Because of the longer runtime of TGFNN, we evaluated 300 combinations for all feature sets besides evaluating 200 on the “All” feature set, due to slower training from the additional features. After evaluating their performance, the combination of hyperparameters with the highest F1 score was selected. Next, we performed five-fold cross-validation to evaluate the performance of the models and datasets on the training and test sets. This trains five instances of each model on a different subset of data, providing information on the variance in performance. To evaluate model calibration, we calibrated the best-performing replicate according to F1 score on the “All” feature set. Each model was calibrated on the training set according to Platt’s method and plotted with the mean probability of ten uniform bins.

### 2.8. Tropical Geometry Fuzzy Neural Network

Due to its lesser-known architecture, we briefly describe the TGFNN, though a full description can be found in [25]. The TGFNN is fuzzy logic classifier built in a neural network architecture that allows flexible and interpretable variable concept encoding, logical rule learning, and inference for a classification task. TGFNN consists of three modules: the encoding module, the rule module, and the inference module.

The encoding module “fuzzifies” continuous input variables into their membership to the concepts “low”, “medium”, and “high”. This encoding is performed via parameterized membership functions that map each variable to three values in the range [0, 1] that represent how much it belongs to each concept. Categorical variables are one-hot encoded. Membership functions are learned during training and help model the intuition and uncertainty in clinician decision making. For example, TGFNN can learn the concept of “low blood pressure” and use that concept in the decision-making rules.

The rule module learns combinations of variable concepts that are predictive of an outcome. The first layer of the rule module learns which concepts are important for each variable within each rule. The second layer learns the importance of each variable within each rule. The more important the variable, the greater the weight within the network, and thus the more it will contribute to inference when activated. Rule activation strength is calculated via a parameterized T-norm which models an AND operation in fuzzy logic via either a product or minimum function. This enables the easily interpretable and logical structure of the decision rules, for example, “if $x_{1}$ is low and $x_{2}$ is high”.

The final layer of the TGFNN is the inference layer, which learns the importance of each rule in determining the model output. The importances, or contributions, of the rules are aggregated in a T-conorm function, followed by softmax activation. This calculates the probability of each output class, given the activation of the rules by the input sample. Implementation of tropical geometry allows the OR operation to be changed between an addition or maximum function.

### 2.9. Statistical Analysis

To evaluate whether differences in model performance across feature sets were significant, we performed Friedman’s tests with the Bonferroni corrected alpha of 0.01 (0.05 divided by the number of tests run, five, one for each metric). For significant Friedman test results, we performed pairwise post hoc Nemenyi tests. We selected these tests because they are non-parametric and recommended when comparing machine learning model cross-validation performance [33].

## 3. Results

We find that, by applying straightforward, interpretable machine learning approaches to EHR data, we are able to predict the onset of first-AMI events in patients without pre-existing cardiovascular conditions, within six months, with moderately good accuracy. Upon comparing different explainable feature engineering approaches (the feature sets), we report that they can have significantly different performance, depending on the model. Overall, the best-performing feature sets were those that included computational phenotypes. Additionally, we compared seven machine and deep learning models, each with a different level of interpretability, and found them to exhibit significantly different performance. We present these results in detail in the following.

### 3.1. Feature Set Performance

**Figure A4:**
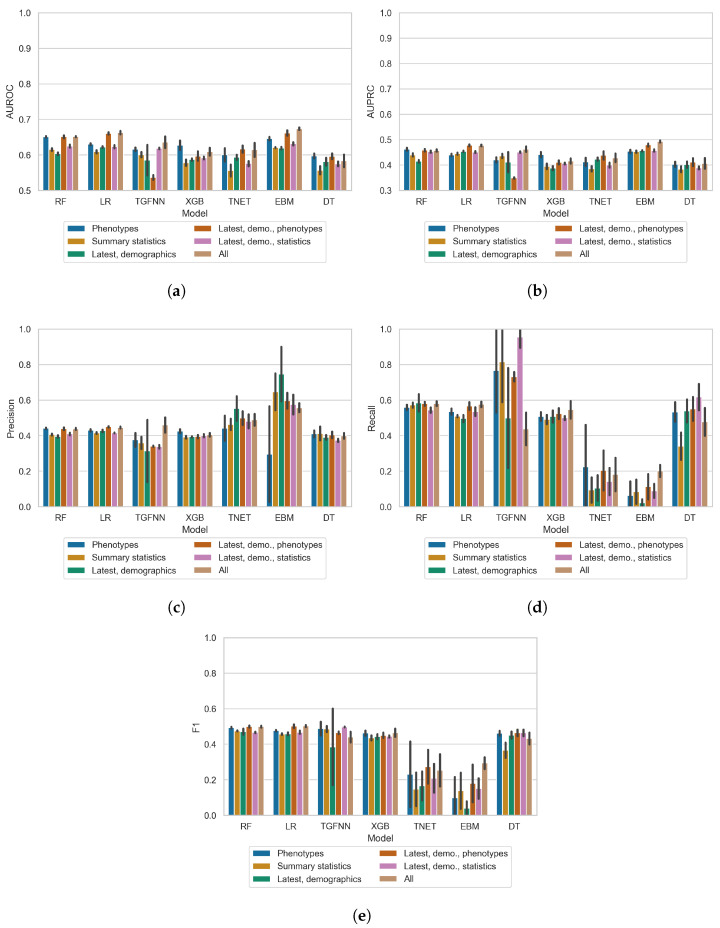
Results of cross-validation-trained models. These plot show the mean test set scores of each model on each feature set. Error bars indicate the standard deviation. The lower limit of the x-axes of the AUROC and AUPRC plots are set to the worst-case scenario of random guessing. (**a**) Mean test AUROC; (**b**) mean test AUPRC; (**c**) mean test precision; (**d**) mean test recall; (**e**) mean test F1.

Feature sets including computational phenotypes significantly outperform those without. Overall, the “All” feature set performs best, though only slightly (see Table 2). This is likely because of the large number of diverse features included, the efficacy of computational phenotyping for feature extraction, and the relevance of historical information. Incorporating computational phenotypes as features resulted in performance gains in AUROC as much as 0.05 (see Table A3 and Figure A4a). We evaluated whether the differences in overall model performance between feature sets were significant by performing Friedman’s test followed by pairwise Nemenyi tests. Across every pairwise comparison, all feature sets containing phenotypes had significantly higher performance than feature sets without phenotypes, according to AUROC. Under AUPRC and F1 score, most of these comparisons were also statistically significant. In no pairwise comparison, regardless of metric, do any of the feature sets containing phenotypes exhibit significantly different performance from each other, save the “All” feature set outperforming the “Phenotypes” feature set according to AUPRC.

### 3.2. Model Performance

We predict the onset of AMI within six months in patients without pre-existing cardiovascular diagnoses with good performance using several interpretable models. Model performance varied significantly between models and feature sets, often depending on the evaluation metric (see Figure A4). While there is no clear “best” model, random forest, logistic regression, and TGFNN performed best overall. In interpreting model performance on an imbalanced dataset, multiple metrics must be appropriately considered as there is no singularly best one. Determining performance criteria is especially important in a clinical application where false positives and negatives could lead to patient harm, either by receiving unnecessary treatment or not receiving needed care, respectively. We note that the models showed varying levels of minimal-to-mild overfitting according to training, validation, and testing set performance (see Table A1, Table A2 and Table A3). When considering all metrics, logistic regression and random forest consistently performed near best, often followed by TGFNN, while XGboost and decision tree were often among the worst. Depending on the metric, TGFNN, EBM, and TabNet typically performed either best or worst (see Figure A4). We performed Friedman tests followed by Nemenyi tests to evaluate whether, across all models, performance differences between feature sets were statistically significant. According to AUROC, random forest, logistic regression, and EBM all performed significantly better than the other models. For nearly all pairwise comparisons, random forest, logistic regression, and TGFNN performed significantly better than XGBoost, decision tree, TabNet, and EBM, according to F1 score. When considering F1 score, there was no significant difference between random forest, logistic regression, and TGFNN performance.

**Figure 1:**
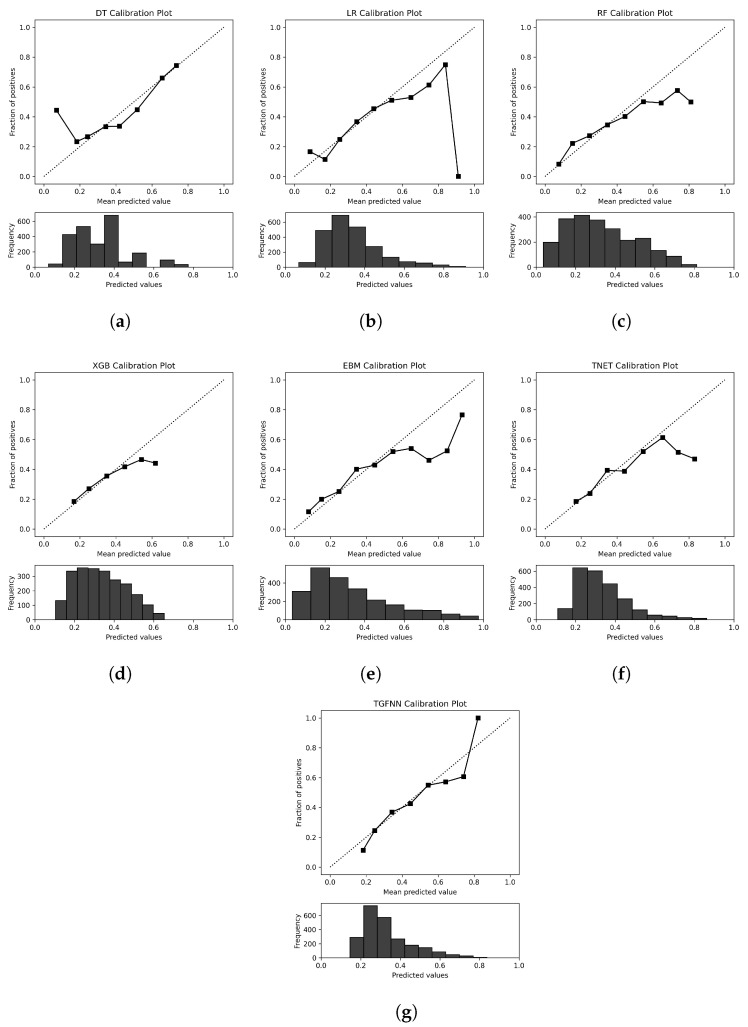
Calibration plots of best model replicate on the “All” feature set, according to F1 score. The diagonal dotted line indicates a classifier with perfect calibration. Samples are grouped into 10 uniformly sized bins, with empty bins excluded. Each point on the curve contrasts the mean model-predicted probability of being a positive sample, with the actual frequency of positive samples, within the bin. (**a**) Decision tree; (**b**) logistic regression; (**c**) random forest; (**d**) XGBoost; (**e**) EBM; (**f**) TabNet; (**g**) TGFNN.

Several models appear biased to over- or underestimating risk of AMI. Across all feature sets, TGFNN exhibits high recall on average (0.754 ± 0.234), as it heavily predicts the positive class. Conversely, TabNet and EBM have low average recall scores (0.159 ± 0.124, 0.095 ± 0.076) due to relatively fewer positive predictions. The accuracy of the, albeit relatively few, positive predictions of EBM contributes to its high mean precision and AUROC (see Table 3). These biases are also present in the model calibration plots (see Figure 1). Both TabNet and EBM underestimate the probability of positive samples while TGFNN slightly overestimates. Overall, the best-performing models, according to F1 score on the “All” feature set, exhibit good calibration.

Several models suffered from variable performance. Model stability is an important factor when considering implementation, especially in a healthcare setting. In contrast to the simpler random forest and logistic regression models, more complicated models, like EBM, TabNetm, and TGFNN, exhibited higher standard deviations in performance (see Figure A4d,e). This may be due to the greater number of hyperparameters that require precise tuning in these models. This variance in performance makes the interpretation of important features difficult as well.

### 3.3. Model Interpretation

Each of the employed models exhibits a degree of inherent interpretability, allowing for some explanation of how predictions were made. For brevity, we will focus our analysis on the “All” feature set, as it is generally the best performing and includes features from all subsets. Additionally, we will focus our interpretability analysis on the better and more consistently performing models: logistic regression, random forest, and XGBoost.

**Figure 2:**
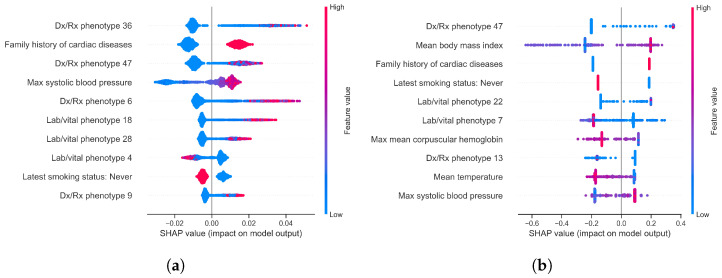
SHAP values of top 10 features, averaged across random forest and XGBoost model replicates trained on the “All” feature set. Each point represents a sample (patient), positioned relative to the impact of the feature on whether the model predicted the positive (AMI) or negative class. The color of points reflect the feature magnitude for the sample. (**a**) Random forest mean SHAP values; (**b**) XGBoost mean SHAP values.

**Figure 3:**
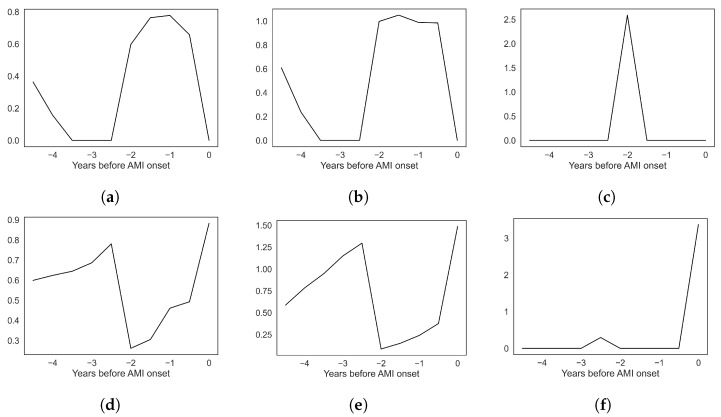
Temporal factors of top six predictive phenotypes. *Y*-axis values are weights learned in tensor decomposition and may give an indication of when the phenotype was characteristically presented. (**a**) Dx/Rx phenotype 47 (back pain); (**b**) Dx/Rx phenotype 36 (cardiometabolic syndrome); (**c**) lab/vital phenotype 18 (ambiguous); (**d**) lab/vital phenotype 28 (kidney disease); (**e**) Dx/Rx phenotype 6 (cardiovascular medication); (**f**) lab/vital phenotype 22 (anemia).

Across all models with global feature importance scores (logistic regression, random forest, XGBoost, EBM, and TabNet), the computational phenotype features of longitudinal EHR data are often the most predictive of a future AMI event. The most important features include Dx/Rx phenotype 47, family history of cardiovascular diseases, Dx/Rx phenotype 36, lab/vital phenotype 18, and a high max systolic blood pressure within the five-year observation window (see Table 4). Feature coefficients in logistic regression and SHAP values of XGBoost and random forest models indicate the direction of the relationship between feature magnitude and future AMI prediction (see Figure 2). The patient phenotypes most strongly indicative of a future AMI are characterized by dorsalgia, type 2 diabetes, hypertension, high creatinine and urea nitrogen levels, cardiovascular medications like atorvastatin, and anemia (see Table 5). The temporal factor of these phenotypes may suggest the characteristic timing of its presentation in patients. We present the temporal factor plots of six of the most predictive phenotypes in Figure 3. The temporal components of the phenotypes predominantly range from immediately before AMI onset to three years prior. Apart from phenotypes being among the most predictive features, additional important variables include a history of smoking and high mean body mass index over the five-year window (Table 6).

**Figure 4:**
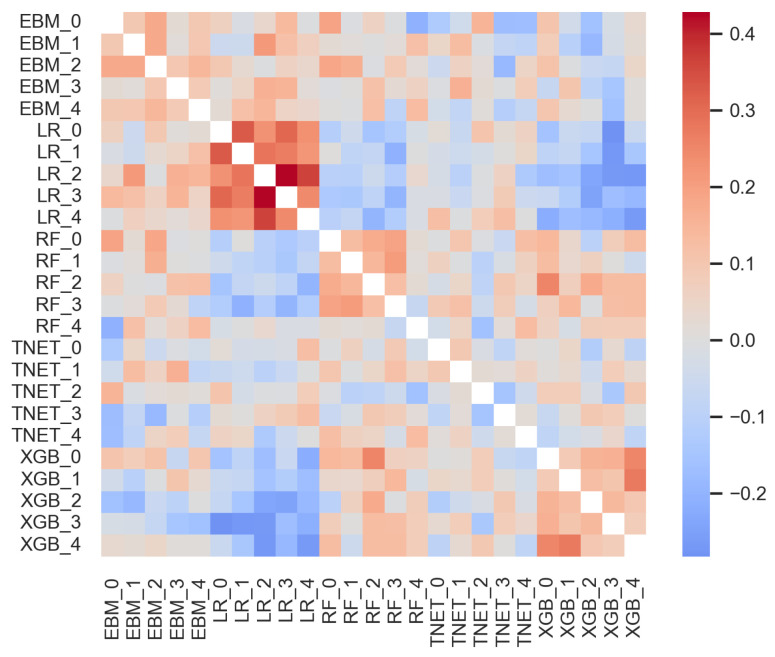
Kendall rank correlation coefficients comparing the ranking of features in the “All” set by importance. A value of 1 indicates perfect positive correlation, −1 is perfect negative correlation, and 0 is no correlation.

The importance of features varies between models, making interpretation difficult at times. When computing the, on average, most important features in the “All” set across all models with global feature importance, there are large standard deviations (see Figure 4). Additionally, we compared how each model cross-validation replicate ranked variables by importance via the Kendall rank correlation coefficient (see Figure 4). This metric shows on a scale of [−1, 1] how negatively or positively correlated the rankings are, with 0 indicating no correlation. We found variable correlation between replicates and models. Logistic regression, random forest, and XGBoost show decent correlation between replicates. On the other hand, TabNet and EBM show relatively low correlation between replicates. Surprisingly, despite somewhat comparable performance, the feature importance ranking of logistic regression is somewhat negatively correlated with the rankings of both random forest and XGBoost. This may be a result of the specific model properties, such as the limitation of logistic regression in identifying linear relationships, whereas XGBoost and random forest can identify nonlinear ones. However, it may also reflect the inherent difficulty in the task of predicting future AMI events in this cohort.

**Figure 5:**
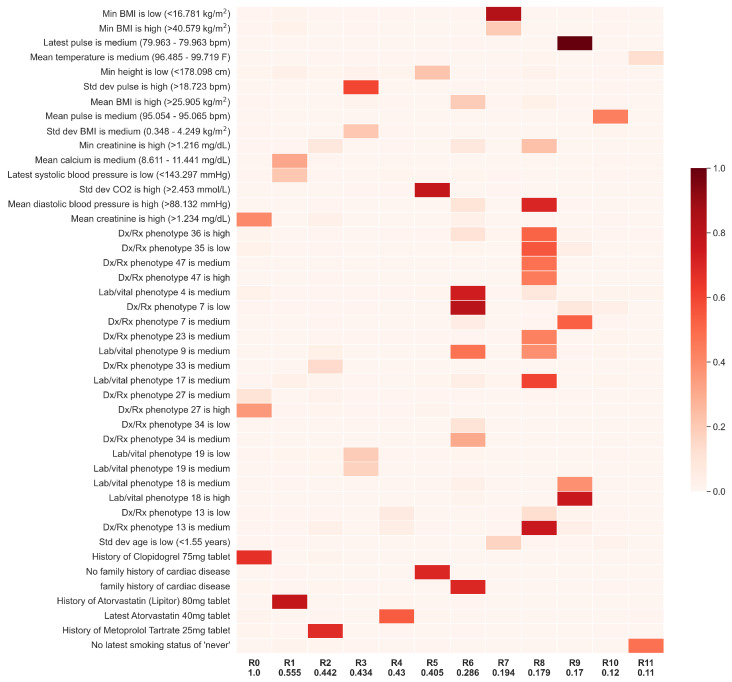
Rules from best-performing TGFNN model on the “All” feature set. The darker red the cell, the more important the concept in the rule. Relative rule contribution to predicting future AMI events is listed along the x-axis. Rules and concepts with less than 0.1 relative importance were removed.

Unlike the other models, TGFNN learns precise, interpretable rules that determine predictions. The best-performing TGFNN model uses the “All” feature set and is based on 12 rules learned directly from the data (AUROC = 0.658, AUPRC = 0.479, F1 = 0.456, precision = 0.475, recall = 0.439). We present these rules in Figure 5. Linguistically, the most important rule (R0) is

Patient has a *history of Clopidogrel prescription* **and** *matches Dx/Rx phenotype 27* (characteristic features include: vitamin D3, simvastatin, vitamin B-12, vitamin C, and malignant neoplasm of bladder (ICD10: C67)) **and** has *high mean creatinine*.

Rules 1–5 are similarly simple to understand, containing a couple of concepts each, and describe combinations of cardiovascular and metabolic medication prescriptions along with abnormal lab and vital measurements, as well as a family history of cardiovascular conditions. Interestingly, while R6 has family history as important, R5 has the lack of family history as important. These rules may be stratifying between different underlying pathologies leading up to AMI. Notably, interpreting the “low”, “medium”, and “high” concepts is dependent on the shape of the underlying membership functions. Because of the flexible, trainable parameters of these functions, they may “squish” the “low” or “high” function out of the possible range of values to dynamically simplify to only two concepts.

Overall, we found the interpretable models able to accurately identify patients, without pre-existing malignant cardiovascular diagnoses, that have an AMI within six months. While the evaluated models varied in both performance and prioritization of important features, we identified several consistently important medical concepts and phenotypes.

## 4. Discussion

We demonstrate that accurately predicting an AMI within six months in patients without pre-existing cardiovascular conditions, using only outpatient data and interpretable models, is possible. Furthermore, we show that temporal, computational phenotyping can identify highly predictive clinical profiles of future AMI events. This suggests the relevance of historical information, temporal EHR relationships, and computational phenotyping in evaluating the future risk of AMI, which is often ignored in similar studies. We anticipate that these findings will be informative to researchers and clinicians seeking to develop interpretable machine learning approaches for hard-to-predict events like AMI, as well as leverage high-dimensional longitudinal EHR data.

The Dx/Rx phenotypes predictive of future AMI onset generally agree with strongly supported clinical relationships and also suggest potential underutilized relationships. The predictive Dx/Rx phenotype 47 describes dorsalgia and other pain as predictive. While low back pain does not have a known association with AMI, chronic pain is associated with various cardiovascular diseases [34,35] and some pain medications, like non-steroidal anti-inflammatory drugs (NSAIDs), are a known risk factor of AMI [36]. The consistently predictive Dx/Rx phenotype 13 describes a profile of pain medication prescriptions, including the NSAID ibuprofen. However, it was negatively related with future AMI according to several models. This discontinuity may be dataset specific or indicate an underlying relationship such as if a patient is on a certain type of medication it reflects their interaction with healthcare professionals that may be helping prevent AMI in other ways. On the other hand, a back pain phenotype may capture patients who are misinterpreting angina (precursor symptom of AMI) for dorsalgia. This could suggest clinicians increase their suspicion of underlying cardiovascular diseases when patients present with back pain. In congruence with known AMI risk factors, Dx/Rx phenotype 36 characterizes patients with type 2 diabetes, potentially further complicated with hypertension [37,38]. The Dx/Rx phenotype number 6 encompasses several cardiovascular medications like the platelet inhibitor clopidogrel. A clopidogrel prescription suggests the patients may have already had severe cardiovascular conditions, like coronary stenosis, that were not recorded in the EHR, that required a stent. This phenotype may be predictive due to poor medication adherence followed by in-stent re-stenosis and a subsequent AMI within approximately six months (see phenotype temporal peak in Figure 3e). However, further analysis is required to ascertain specific and supported claims of this clinical relationship.

The lab/vital phenotypes suggest some clinically valid risk factors, but are noticeably harder to interpret due to large quantile ranges. Lab/vital phenotype 28 characterizes patients with mild-to-severe kidney disease, indicated by elevated creatinine [39], high urea nitrogen [40], and hyperchloremia [41]. Kidney disease greatly increases the risk of adverse cardiac events like AMI [42]. Lab/vital phenotype 22 describes a patient with mild-to-severe anemia, a risk factor of AMI [43]. The temporal component of the phenotype suggests that this occurs relatively soon before AMI (see Figure 3f). However, the large range of these lab result quantiles limits the utility of the phenotypes. In the future, more precise partitioning of variables may resolve this. Notably, lab/vital phenotype 18 does not described abnormal physiology. The range of the “Absolute Early Granulocyte Count” encompasses essentially all possible values. A deeper look at the distribution of values in the training data suggested this is a result of too few unique values to make five equally sized quantiles. Additionally, the feature weights in this phenotype are relatively low, indicating weak membership and thus a rather ambiguous phenotype. The relevance of this phenotype with future AMI may be an artifact of the data or methods. While phenotypes using laboratory values and vital signs can be improved, they can successfully capture important abnormal physiology across temporal EHRs.

Visualizing SHAP values of the latest value and summary statistic features revealed additional risk factors with known clinical relevance. Unlike standard feature importance scores generated by tree-based models, SHAP values indicate the direction of relationships between features and outcomes. Specifically, the SHAP values of the random forest and XGBoost models trained on the “All” feature set suggest several predictive relationships (see Figure 2). These predictive variables include high blood pressure [44], family history of cardiovascular diseases [45], high body mass index [12], smoking [46], and low mean corpuscular hemoglobin levels [47]. Additionally, the SHAP values agree with other feature importance scores, indicating the presence of Dx/Rx phenotypes 36 and 47, back pain and cardiometabolic syndrome, respectively, are predictive of a future AMI. Other features were not consistently highly predictive across multiple replicates or models. The contradictions and variability in the importance of features and their relationship to future AMI events are likely a result of noise within the used EHR data and reflect a typical challenge in predictive machine learning in healthcare. Computational approaches, such as this work, may best serve as a screening method for a specific clinical relationship to be explored in more controlled settings.

When compared to summary statistics and the most recent recorded data, computational EHR phenotypes can significantly increase interpretability and performance in biomedical machine learning. Across multiple model architectures and feature sets, the phenotypes consistently ranked as the most important features. These results suggest that historical and temporal information encoded in EHRs is highly relevant for predictive modeling, and specifically for AMI risk assessment. Additionally, we suggest the increased use of tensor decomposition in EHR feature extraction. The employed tensor factorization algorithm mines temporal, high-dimensional EHR data without supervision, removing the need for clinicians to manually curate phenotypes. These phenotypes capture patterns of co-occurring medical variables across time to describe distinct patient profiles. These patient phenotypes reduce the dimensionality of the EHR data while maintaining interpretability and improving performance. Predictive phenotypes can prioritize to clinicians the important sets of conditions patients present with in the clinic, that may be indicative of risk for a future AMI. These can direct more targeted studies to establish association. Additionally, they can provide information regarding the timing of conditions, which may prompt further investigation into understanding the progression and evolution of disease, as well as potential timing for early intervention. Notably, computational phenotypes may be difficult to interpret if they are redundant, have many features with similar weight, or do not make clinical sense. Many improvements upon the base PARAFAC tensor factorization have been made to address these problems specifically for temporal EHR phenotyping [24]. However, in this work, we focused on the baseline approach due to its wide accessibility.

While we did not identify a singular superior, interpretable machine learning model, we identified several strengths and weaknesses. Overall, random forest, logistic regression, and TGFNN performed best. All models exhibit good calibration. In a similar AMI prediction study, [11] presents poor model calibration results. As the authors state, poor calibration in [11] is likely a result of very severe class imbalance, whereas in this work we limit class imbalance via the downsampling of matched negative samples and class-weighted loss functions. Some models, like EBM and TabNet, displayed very poor recall and F1 scores due to biased class predictions. They also showed low concordance in feature importance between cross-validation instances. It is likely that these models were not well suited for this particular dataset and task. The TGFNN provides clear rules for predictions, making it perhaps the most interpretable of the models. The rule-based nature of TGFNN well reflects how clinicians make decisions and identify patterns. We anticipate that the further development of TGFNN and other interpretable rule-based models will aid clinical adoption. Still, in the example presented in Figure 5, interpretation can be difficult if “medium” concepts cannot be clarified. Logistic regression and random forest both showed some of the best performance and consistency of feature importance. These models are often too simplistic to solve difficult tasks; however, in this case, deriving features from computational phenotypes improved performance.

This study has several limitations that affect the applicability and bias of results. First, the employed cohort of patients comes from a single hospital system and is predominantly elderly and white. We excluded data on procedures received by patients. We employed mRMR feature selection, which may not find the optimal set of features. Additionally, the interpretation of important features showed high variability between and even within models. We note that while similar studies attempting to predict AMI, such as the work carried out in [11], show higher AUROC values, this work attempts a potentially more difficult task to predict AMI events within a cohort without pre-existing cardiac conditions. Future work could address these limitations by expanding the cohort inclusion criteria, incorporating data from multiple healthcare systems, as well as using computational methods to explore the causal relationships between clinical features and AMI onset.

In conclusion, we suggest that temporal, computational phenotyping can improve the utility of outpatient EHRs in both predicting the risk of AMI in otherwise low-risk patients and identifying novel risk factors for further investigation. Additionally, we demonstrate that interpretable machine learning models can consistently identify important risk factors and accurately predict a future AMI event in patients without pre-existing cardiovascular conditions, using only outpatient data. We note that model-derived feature importance scores may be discordant, and encourage researchers to validate findings. We anticipate that these findings will promote further development in computational and machine learning approaches to identify novel phenotypes that can aid clinicians in understanding, predicting, and preventing AMI and subsequent hospitalization.

## Figures and Tables

**Table 1:** Overview of cohort. Age and hospital frailty risk score are presented as mean ± standard deviation. All percentages are of the patients within the column subset, besides the “Number of patients” which is the percentage of positive/negative patients within in the train/test set.

	Train		Test	
	Control	Cases	Controls	Cases
Number of patients	3705 (66.5%)	1865 (33.5%)	1582 (67.1%)	776 (32.9%)
Age, years	63.5 ± 13.0	63.6 ± 2.9	63.5 ± 2.9	63.8 ± 12.9
Hospital frailty risk score	4.6 ± 6.1	4.9 ± 6.2	4.7 ± 6.0	4.9 ± 6.4
Sex, male	2093 (56.5%)	1092 (58.6%)	918 (58.0%)	433 (55.8%)
Cardiac family history	1524 (41.1%)	971 (52.1%)	644 (40.7%)	378 (48.7%)
**Ethnicity**				
White or Caucasian	3154 (85.1%)	1553 (83.3%)	1347 (85.1%)	671 (86.5%)
Black or African American	248 (6.7%)	164 (8.8%)	111 (7.0%)	61 (7.9%)
Asian	133 (3.6%)	54 (2.9%)	68 (4.3%)	16 (2.1%)
Other	97 (2.6%)	49 (2.6%)	32 (2.0%)	13 (1.7%)
Unknown	33 (0.9%)	21 (1.1%)	14 (0.9%)	8 (1.0%)
American Native	11 (0.3%)	12 (0.6%)	2 (0.1%)	5 (0.6%)
Native Pacific Islander	3 (0.1%)	2 (0.1%)	1 (0.1%)	0 (0.0%)

**Table 2:** Mean ± standard deviation scores for each feature set across all models, sorted by F1 score.

Feature Set	AUROC	AUPRC	F1	Precision	Recall
All	**0.63 ± 0.03**	**0.45 ± 0.03**	**0.41 ± 0.10**	0.46 ± 0.06	0.43 ± 0.17
Latest, demo., phenotypes	0.62 ± 0.04	0.43 ± 0.04	0.4 ± 0.13	0.45 ± 0.08	0.47 ± 0.22
Phenotypes	0.62 ± 0.02	0.43 ± 0.02	0.39 ± 0.17	0.4 ± 0.11	0.46 ± 0.25
Latest, demo., statistics	0.61 ± 0.02	0.43 ± 0.03	0.39 ± 0.14	0.43 ± 0.08	**0.48 ± 0.28**
Summary statistics	0.59 ± 0.03	0.42 ± 0.03	0.36 ± 0.15	0.44 ± 0.1	0.42 ± 0.26
Latest, demographics	0.6 ± 0.02	0.42 ± 0.03	0.35 ± 0.18	**0.46 ± 0.16**	0.39 ± 0.24

**Table 3:** Mean ± standard deviation scores for each model, across all feature sets, sorted by F1 score.

Model	AUROC	AUPRC	F1	Precision	Recall
RF	0.633 ± 0.02	0.448 ± 0.017	**0.484 ± 0.015**	0.422 ± 0.019	0.57 ± 0.026
LR	0.635 ± 0.021	0.458 ± 0.015	0.478 ± 0.02	0.431 ± 0.014	0.537 ± 0.034
TGFNN	0.599 ± 0.037	0.422 ± 0.041	0.46 ± 0.091	0.365 ± 0.085	**0.701 ± 0.246**
XGBoost	0.598 ± 0.019	0.41 ± 0.019	0.45 ± 0.017	0.402 ± 0.014	0.513 ± 0.034
DT	0.582 ± 0.017	0.4 ± 0.016	0.44 ± 0.043	0.399 ± 0.023	0.51 ± 0.108
TabNET	0.593 ± 0.025	0.415 ± 0.022	0.213 ± 0.111	0.488 ± 0.057	0.159 ± 0.124
EBM	**0.642 ± 0.021**	**0.466 ± 0.016**	0.15 ± 0.11	**0.57 ± 0.187**	0.095 ± 0.076

**Table 4:** Top 10 most important features from the “All” feature set, reported as min–max scaled mean ± standard deviation of all LR, RF, XGB, EBM, and TNET models.

Feature	Importance
Dx/Rx phenotype 47	0.533 ± 0.316
Family history of cardiovascular diseases	0.511 ± 0.324
Dx/Rx phenotype 36	0.498 ± 0.374
Lab/vital phenotype 18	0.496 ± 0.304
Max systolic blood pressure	0.44 ± 0.338
Dx/Rx phenotype 13	0.405 ± 0.216
Lab/vital phenotype 4	0.404 ± 0.256
Lab/vital phenotype 28	0.359 ± 0.252
Lab/vital phenotype 9	0.342 ± 0.195
Lab/vital phenotype 11	0.336 ± 0.245

**Table 5:** Phenotypes most predictive of impending AMI, based on logistic regression coefficients and SHAP values from random forest and XGBoost. The top five features of each are shown. Units: millimoles per liter (mmol/L), milligrams per deciliter (mg/dL), grams per deciliter (g/dL), multiples of a thousand per cubic millimeter (K/mm^3^), multiples of a million per cubic millimeter (M/mm^3^).

Dx/Rx Phenotype 47 (Back Pain)	Weight
Dorsalgia (M54)	0.0252
Low back pain (M545)	0.0148
Other joint disorder, not elsewhere classified (M25)	0.0118
Pain, not elsewhere classified (G89)	0.0111
Other chronic pain (G8929)	0.0097
**Dx/Rx Phenotype 36 (cardiometabolic syndrome)**	**Weight**
Type 2 diabetes mellitus (E11)	0.0463
Type 2 diabetes mellitus without complications (E119)	0.0413
Essential (primary) hypertension (I10)	0.0113
Type 2 diabetes mellitus with hyperglycemia (E1165)	0.0076
Disorders of lipoprotein metabolism and other lipidemias (E78)	0.0064
**Lab/vital Phenotype 18 (ambiguous)**	**Weight**
Absolute Basophil Count, (−0.001, 0.1] K/mm^3^	0.0017
Absolute Early Granulocyte Count, (−0.001, 13.7] K/mm^3^	0.0017
Potassium, (4.0, 4.3] mmol/L	0.0009
Chloride, (104.0, 106.0] mmol/L	0.0009
Urea Nitrogen, (4.999, 13.0] mg/dL	0.0009
**Lab/vital Phenotype 28 (kidney disease)**	**Weight**
Creatinine, (1.122, 21.48] mg/dL	0.1908
Urea Nitrogen, (23.0, 139.0] mg/dL	0.1631
CO_2_, (9.999, 26.0] mmol/L	0.0980
Chloride, (107.0, 122.0] mmol/L	0.0663
Potassium, (4.7, 9.8] mmol/L	0.0550
**Dx/Rx Phenotype 6 (cardiovascular medication)**	**Weight**
Atorvastatin 40 mg tablet	0.0214
Amlodipine 10 mg tablet	0.0095
Clopidogrel 75 mg tablet	0.0050
Lisinopril 40 mg tablet	0.0025
Metoprolol succinate ER 25 mg 24 h tablet	0.0016
**Lab/vital Phenotype 22 (anemia)**	**Weight**
Hemoglobin, (5.699, 12.1] g/dL	0.0559
Hematocrit, (18.099, 36.5] %	0.0547
Red Blood Cell Count, (1.87, 4.02] M/mm^3^	0.0517
Calcium, (4.799, 9.2] mg/dL	0.0512
Albumin, (1.499, 4.0] g/dL	0.0501

**Table 6:** Top 10 logistic regression feature coefficients from the best-performing model on the “All” feature set, reported as mean ± standard deviation. Milligrams (mg).

Feature	Coefficient
Dx/Rx phenotype 7	−1.346 ± 0.191
Lab/vital phenotype 4	−0.911 ± 0.08
Lab/vital phenotype 18	0.858 ± 0.154
Lab/vital phenotype 11	−0.79 ± 0.046
Clopidogrel, 75 mg table (within last 5 years)	0.777 ± 0.076
Dx/Rx phenotype 47	0.768 ± 0.379
Dx/Rx phenotype 46	−0.712 ± 0.156
Dx/Rx phenotype 13	−0.708 ± 0.089
Lab/vital phenotype 9	−0.69 ± 0.106
Dx/Rx phenotype 35	−0.626 ± 0.221

**Figure A2:**
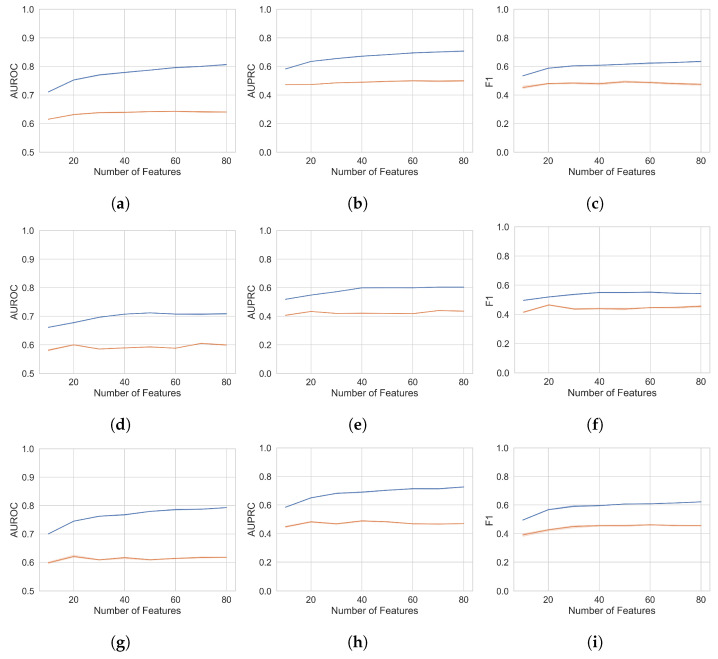
The predictive performance of random forest using different-sized feature sets selected by mRMR. The lines (blue = training set, orange = validation set) show the mean score of three replicates, with shaded regions showing 95% confidence intervals. (**a**) AUROC for phenotypes; (**b**) AUPRC for phenotypes; (**c**) F1 for phenotypes; (**d**) AUROC for latest, demo.; (**e**) AUPRC for latest, demo.; (**f**) F1 for latest, demo.; (**g**) AUROC for summary statistics; (**h**) AUPRC for summary statistics; (**i**) F1 for summary statistics.

**Figure A3:**
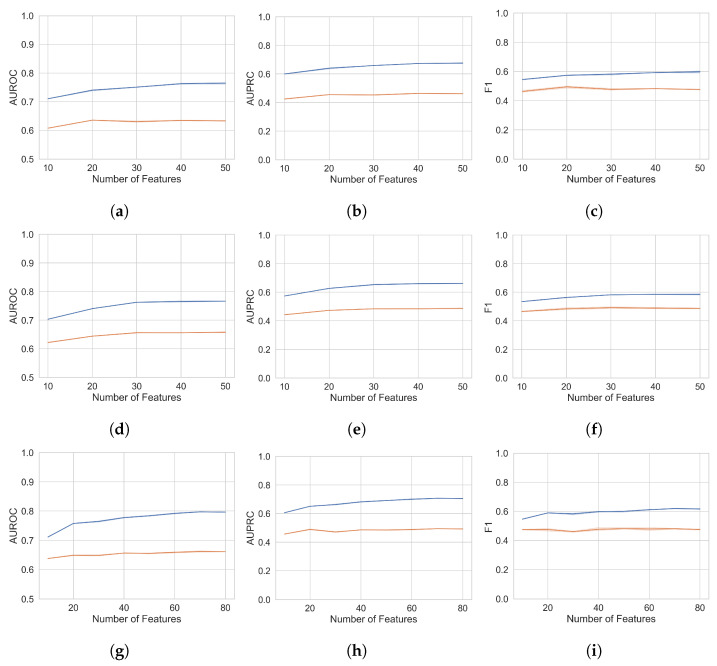
The predictive performance of random forest using different-sized feature sets selected by mRMR. The lines (blue = training set, orange = validation set) show the mean score of three replicates, with shaded regions showing 95% confidence intervals. (**a**) AUROC for latest, demo., statistics; (**b**) AUPRC for latest, demo., statistics; (**c**) F1 for latest, demo., statistics; (**d**) AUROC for latest, demo., phenotypes; (**e**) AUPRC for latest, demo., phenotypes; (**f**) F1 for latest, demo., phenotypes; (**g**) AUROC for All; (**h**) AUPRC for All; (**i**) F1 for All.

## Data Availability

The datasets generated and/or analyzed during the current study were collected at Michigan Medicine. The University of Michigan’s Innovation Partnerships (UMIP) unit will handle potential charges/arrangements of the use of data by external entities, using such methods as material transfer agreements. Please contact UMIP (innovationpartnerships@umich.edu) for data inquiries.

## Abbreviations

The following abbreviations are used in this manuscript:

AMI	Acute Myocardial Infarction
EHR	Electronic Health Record
LR	Logistic Regression
RF	Random Forest
TGFNN	Tropical Geometry Fuzzy Neural Network
EBM	Explainable Boosting Machine
XGBoost	eXtreme Gradient Boosting
XGB	eXtreme Gradient Boosting
TNET	TabNet
AUROC	Area Under the Receiver Operating Characteristic Curve
AUPRC	Area Under the Precision Recall Curve
mRMR	Minimum Redundancy Maximum Relevance
ICD10	10th revision of the International Statistical Classification of Diseases
SHAP	SHapley Additive exPlanations
NSAID	Non-Steroidal Anti-Inflammatory Drug

## Appendix A

**Table A1:** Model cross-validation results on training set.

Feature Set	Model	AUROC	AUPRC	F1	Precision	Recall
	DT	0.682 ± 0.01	0.514 ± 0.024	0.526 ± 0.023	0.488 ± 0.038	0.577 ± 0.061
	EBM	**0.796 ± 0.011**	**0.669 ± 0.014**	0.418 ± 0.047	**0.776 ± 0.016**	0.288 ± 0.045
	LR	0.677 ± 0.01	0.512 ± 0.011	0.528 ± 0.009	0.479 ± 0.01	0.588 ± 0.009
All	RF	0.729 ± 0.004	0.562 ± 0.006	**0.575 ± 0.004**	0.505 ± 0.005	**0.666 ± 0.006**
	TGFNN	0.647 ± 0.027	0.489 ± 0.025	0.458 ± 0.04	0.478 ± 0.048	0.455 ± 0.096
	TNET	0.672 ± 0.019	0.513 ± 0.05	0.318 ± 0.097	0.611 ± 0.018	0.224 ± 0.094
	XGB	0.682 ± 0.006	0.511 ± 0.005	0.526 ± 0.009	0.462 ± 0.006	0.612 ± 0.024
	DT	0.712 ± 0.011	0.547 ± 0.011	0.557 ± 0.009	0.484 ± 0.029	0.664 ± 0.064
	EBM	**0.736 ± 0.017**	**0.591 ± 0.022**	0.232 ± 0.134	**0.783 ± 0.123**	0.146 ± 0.092
	LR	0.655 ± 0.011	0.482 ± 0.011	0.505 ± 0.012	0.458 ± 0.014	0.562 ± 0.009
Latest, demo., phenotypes	RF	0.698 ± 0.005	0.529 ± 0.006	**0.545 ± 0.001**	0.475 ± 0.004	0.638 ± 0.005
	TGFNN	0.537 ± 0.008	0.36 ± 0.005	0.464 ± 0.008	0.343 ± 0.003	**0.717 ± 0.031**
	TNET	0.674 ± 0.015	0.511 ± 0.039	0.331 ± 0.109	0.596 ± 0.009	0.241 ± 0.117
	XGB	0.652 ± 0.01	0.469 ± 0.014	0.506 ± 0.008	0.445 ± 0.008	0.586 ± 0.017
	DT	0.65 ± 0.01	0.477 ± 0.008	0.526 ± 0.011	0.429 ± 0.02	0.686 ± 0.071
	EBM	0.715 ± 0.013	0.568 ± 0.018	0.19 ± 0.094	**0.771 ± 0.05**	0.113 ± 0.063
	LR	0.635 ± 0.01	0.475 ± 0.012	0.48 ± 0.005	0.438 ± 0.01	0.532 ± 0.007
Latest, demo., statistics	RF	0.708 ± 0.007	0.559 ± 0.004	0.545 ± 0.005	0.489 ± 0.012	0.617 ± 0.01
	TGFNN	0.634 ± 0.015	0.479 ± 0.017	0.506 ± 0.006	0.344 ± 0.012	**0.96 ± 0.052**
	TNET	0.616 ± 0.02	0.458 ± 0.058	0.246 ± 0.093	0.563 ± 0.016	0.164 ± 0.084
	XGB	**0.716 ± 0.01**	**0.568 ± 0.008**	**0.549 ± 0.013**	0.502 ± 0.012	0.607 ± 0.021
	DT	0.652 ± 0.012	0.489 ± 0.021	**0.508 ± 0.018**	0.445 ± 0.018	0.595 ± 0.063
	EBM	0.646 ± 0.018	0.49 ± 0.019	0.051 ± 0.052	**0.867 ± 0.126**	0.027 ± 0.028
	LR	0.632 ± 0.016	0.476 ± 0.019	0.469 ± 0.019	0.44 ± 0.013	0.504 ± 0.027
Latest, demographics	RF	0.627 ± 0.013	0.449 ± 0.013	0.504 ± 0.01	0.423 ± 0.012	**0.622 ± 0.017**
	TGFNN	0.583 ± 0.035	0.402 ± 0.028	0.381 ± 0.213	0.311 ± 0.174	0.493 ± 0.277
	TNET	**0.655 ± 0.017**	**0.493 ± 0.048**	0.193 ± 0.102	0.673 ± 0.024	0.119 ± 0.08
	XGB	0.613 ± 0.015	0.421 ± 0.013	0.465 ± 0.012	0.421 ± 0.013	0.519 ± 0.026
	DT	0.684 ± 0.012	0.498 ± 0.017	0.542 ± 0.007	0.482 ± 0.035	0.625 ± 0.057
	EBM	0.726 ± 0.033	**0.579 ± 0.044**	0.158 ± 0.162	**0.85 ± 0.116**	0.099 ± 0.111
	LR	0.631 ± 0.018	0.456 ± 0.018	0.484 ± 0.009	0.442 ± 0.018	0.535 ± 0.02
Phenotypes	RF	0.713 ± 0.01	0.559 ± 0.01	0.552 ± 0.007	0.497 ± 0.007	0.621 ± 0.012
	TGFNN	0.628 ± 0.013	0.443 ± 0.011	0.498 ± 0.029	0.384 ± 0.042	**0.776 ± 0.223**
	TNET	0.631 ± 0.028	0.457 ± 0.061	0.276 ± 0.201	0.531 ± 0.033	0.242 ± 0.235
	XGB	**0.726 ± 0.012**	0.568 ± 0.021	**0.563 ± 0.013**	0.514 ± 0.016	0.623 ± 0.011
	DT	0.664 ± 0.006	0.512 ± 0.011	0.462 ± 0.032	0.531 ± 0.06	0.421 ± 0.073
	EBM	0.717 ± 0.031	**0.575 ± 0.043**	0.162 ± 0.13	**0.846 ± 0.099**	0.096 ± 0.084
	LR	0.629 ± 0.01	0.478 ± 0.012	0.464 ± 0.007	0.44 ± 0.014	0.492 ± 0.004
Summary statistics	RF	0.711 ± 0.004	0.567 ± 0.005	0.555 ± 0.005	0.486 ± 0.007	0.646 ± 0.01
	TGFNN	0.633 ± 0.02	0.477 ± 0.023	0.506 ± 0.004	0.381 ± 0.056	**0.828 ± 0.212**
	TNET	0.592 ± 0.013	0.435 ± 0.077	0.164 ± 0.099	0.592 ± 0.043	0.1 ± 0.069
	XGB	**0.721 ± 0.009**	0.572 ± 0.008	**0.559 ± 0.006**	0.512 ± 0.011	0.615 ± 0.003

**Table A2:** Model cross-validation results on validation set.

Feature Set	Model	AUROC	AUPRC	F1	Precision	Recall
	DT	0.596 ± 0.033	0.422 ± 0.042	0.444 ± 0.029	0.424 ± 0.045	0.479 ± 0.078
	EBM	**0.671 ± 0.043**	**0.508 ± 0.061**	0.292 ± 0.034	**0.622 ± 0.13**	0.197 ± 0.041
	LR	0.634 ± 0.032	0.469 ± 0.05	0.483 ± 0.045	0.448 ± 0.039	0.534 ± 0.089
All	RF	0.643 ± 0.045	0.466 ± 0.06	**0.495 ± 0.016**	0.45 ± 0.046	**0.56 ± 0.054**
	TGFNN	0.614 ± 0.042	0.457 ± 0.053	0.42 ± 0.077	0.441 ± 0.039	0.42 ± 0.138
	TNET	0.566 ± 0.018	0.387 ± 0.224	0.156 ± 0.108	0.442 ± 0.252	0.1 ± 0.079
	XGB	0.583 ± 0.025	0.414 ± 0.034	0.439 ± 0.042	0.396 ± 0.028	0.504 ± 0.099
	DT	0.586 ± 0.028	0.414 ± 0.03	0.452 ± 0.029	0.405 ± 0.038	0.522 ± 0.085
	EBM	**0.642 ± 0.034**	**0.469 ± 0.053**	0.165 ± 0.104	**0.518 ± 0.125**	0.106 ± 0.073
	LR	0.632 ± 0.039	0.465 ± 0.053	0.48 ± 0.049	0.447 ± 0.049	0.528 ± 0.085
Latest, demo., phenotypes	RF	0.64 ± 0.048	0.464 ± 0.06	**0.496 ± 0.023**	0.443 ± 0.049	0.571 ± 0.049
	TGFNN	0.532 ± 0.014	0.357 ± 0.007	0.46 ± 0.026	0.339 ± 0.009	**0.718 ± 0.086**
	TNET	0.576 ± 0.02	0.39 ± 0.226	0.156 ± 0.111	0.467 ± 0.272	0.098 ± 0.072
	XGB	0.606 ± 0.024	0.425 ± 0.026	0.467 ± 0.023	0.412 ± 0.019	0.546 ± 0.069
	DT	0.596 ± 0.03	0.411 ± 0.022	0.485 ± 0.033	0.395 ± 0.026	0.633 ± 0.075
	EBM	**0.641 ± 0.041**	**0.487 ± 0.048**	0.152 ± 0.045	**0.641 ± 0.128**	0.088 ± 0.031
	LR	0.619 ± 0.039	0.464 ± 0.05	0.464 ± 0.049	0.427 ± 0.034	0.517 ± 0.096
Latest, demo., statistics	RF	0.634 ± 0.039	0.472 ± 0.052	0.474 ± 0.04	0.425 ± 0.032	0.543 ± 0.081
	TGFNN	0.615 ± 0.037	0.461 ± 0.05	**0.507 ± 0.009**	0.344 ± 0.013	**0.962 ± 0.05**
	TNET	0.562 ± 0.022	0.391 ± 0.229	0.146 ± 0.086	0.472 ± 0.282	0.087 ± 0.054
	XGB	0.594 ± 0.013	0.431 ± 0.022	0.437 ± 0.019	0.397 ± 0.007	0.488 ± 0.044
	DT	0.575 ± 0.03	0.398 ± 0.03	**0.456 ± 0.032**	0.4 ± 0.03	0.535 ± 0.074
	EBM	0.617 ± 0.059	0.46 ± 0.072	0.025 ± 0.036	**0.556 ± 0.37**	0.013 ± 0.02
	LR	**0.618 ± 0.063**	**0.464 ± 0.075**	0.444 ± 0.1	0.42 ± 0.048	0.495 ± 0.168
Latest, demographics	RF	0.602 ± 0.048	0.423 ± 0.054	0.454 ± 0.095	0.399 ± 0.039	**0.551 ± 0.177**
	TGFNN	0.57 ± 0.051	0.391 ± 0.049	0.353 ± 0.217	0.298 ± 0.168	0.476 ± 0.352
	TNET	0.556 ± 0.029	0.385 ± 0.228	0.106 ± 0.074	0.513 ± 0.298	0.06 ± 0.042
	XGB	0.584 ± 0.047	0.4 ± 0.046	0.45 ± 0.05	0.396 ± 0.052	0.531 ± 0.086
	DT	0.584 ± 0.048	0.408 ± 0.047	0.462 ± 0.053	0.411 ± 0.035	0.53 ± 0.089
	EBM	0.632 ± 0.054	0.462 ± 0.065	0.106 ± 0.111	0.428 ± 0.26	0.07 ± 0.084
	LR	0.603 ± 0.065	0.434 ± 0.071	0.465 ± 0.046	0.42 ± 0.059	0.523 ± 0.037
Phenotypes	RF	**0.64 ± 0.064**	**0.475 ± 0.078**	0.493 ± 0.035	**0.445 ± 0.062**	0.559 ± 0.028
	TGFNN	0.605 ± 0.064	0.426 ± 0.069	**0.5 ± 0.042**	0.393 ± 0.057	**0.772 ± 0.235**
	TNET	0.547 ± 0.024	0.363 ± 0.331	0.069 ± 0.073	0.381 ± 0.35	0.038 ± 0.041
	XGB	0.607 ± 0.045	0.444 ± 0.066	0.47 ± 0.018	0.427 ± 0.037	0.528 ± 0.032
	DT	0.552 ± 0.02	0.382 ± 0.012	0.357 ± 0.044	0.408 ± 0.06	0.336 ± 0.084
	EBM	**0.631 ± 0.046**	**0.477 ± 0.048**	0.126 ± 0.101	**0.685 ± 0.209**	0.078 ± 0.07
	LR	0.605 ± 0.04	0.454 ± 0.047	0.447 ± 0.017	0.418 ± 0.036	0.484 ± 0.029
Summary statistics	RF	0.613 ± 0.041	0.454 ± 0.049	0.465 ± 0.02	0.404 ± 0.035	0.553 ± 0.051
	TGFNN	0.602 ± 0.044	0.447 ± 0.047	**0.484 ± 0.031**	0.353 ± 0.018	**0.817 ± 0.223**
	TNET	0.544 ± 0.021	0.361 ± 0.33	0.051 ± 0.049	0.44 ± 0.403	0.027 ± 0.026
	XGB	0.591 ± 0.02	0.432 ± 0.023	0.437 ± 0.024	0.4 ± 0.027	0.485 ± 0.049

**Table A3:** Model cross-validation results on test set.

Feature Set	Model	AUROC	AUPRC	F1	Precision	Recall
	DT	0.583 ± 0.018	0.407 ± 0.021	0.432 ± 0.033	0.4 ± 0.016	0.478 ± 0.079
	EBM	**0.674 ± 0.003**	**0.494 ± 0.004**	0.294 ± 0.033	**0.558 ± 0.025**	0.202 ± 0.034
	LR	0.663 ± 0.005	0.478 ± 0.003	**0.503 ± 0.006**	0.446 ± 0.005	0.576 ± 0.016
All	RF	0.652 ± 0.002	0.458 ± 0.004	0.5 ± 0.005	0.439 ± 0.006	**0.581 ± 0.014**
	TGFNN	0.636 ± 0.016	0.463 ± 0.01	0.441 ± 0.03	0.46 ± 0.043	0.438 ± 0.092
	TNET	0.615 ± 0.02	0.429 ± 0.017	0.253 ± 0.091	0.49 ± 0.033	0.182 ± 0.094
	XGB	0.609 ± 0.011	0.416 ± 0.01	0.465 ± 0.023	0.405 ± 0.01	0.546 ± 0.049
	DT	0.596 ± 0.008	0.412 ± 0.016	0.464 ± 0.018	0.404 ± 0.018	0.551 ± 0.067
	EBM	0.662 ± 0.007	**0.479 ± 0.006**	0.18 ± 0.106	**0.597 ± 0.045**	0.113 ± 0.071
	LR	0.661 ± 0.003	0.478 ± 0.004	**0.502 ± 0.01**	0.45 ± 0.003	0.567 ± 0.022
Latest, demo., phenotypes	RF	**0.651 ± 0.004**	0.459 ± 0.005	0.5 ± 0.005	0.439 ± 0.007	0.58 ± 0.011
	TGFNN	0.537 ± 0.007	0.349 ± 0.003	0.465 ± 0.007	0.341 ± 0.003	**0.732 ± 0.026**
	TNET	0.617 ± 0.01	0.438 ± 0.016	0.274 ± 0.095	0.499 ± 0.039	0.204 ± 0.113
	XGB	0.597 ± 0.013	0.411 ± 0.008	0.45 ± 0.015	0.395 ± 0.01	0.524 ± 0.031
	DT	0.575 ± 0.007	0.389 ± 0.006	0.464 ± 0.019	0.374 ± 0.009	0.618 ± 0.074
	EBM	**0.631 ± 0.004**	**0.458 ± 0.004**	0.151 ± 0.058	**0.575 ± 0.056**	0.089 ± 0.04
	LR	0.623 ± 0.004	0.452 ± 0.004	0.468 ± 0.009	0.416 ± 0.002	0.536 ± 0.025
Latest, demo., statistics	RF	0.625 ± 0.004	0.453 ± 0.005	0.467 ± 0.004	0.41 ± 0.007	0.544 ± 0.015
	TGFNN	0.619 ± 0.002	0.452 ± 0.003	**0.498 ± 0.002**	0.337 ± 0.01	**0.955 ± 0.06**
	TNET	0.575 ± 0.007	0.4 ± 0.01	0.209 ± 0.081	0.481 ± 0.039	0.142 ± 0.077
	XGB	0.591 ± 0.004	0.407 ± 0.003	0.444 ± 0.006	0.4 ± 0.009	0.5 ± 0.012
	DT	0.581 ± 0.011	0.402 ± 0.013	0.451 ± 0.02	0.391 ± 0.013	0.539 ± 0.065
	EBM	0.619 ± 0.003	**0.456 ± 0.003**	0.04 ± 0.039	**0.747 ± 0.153**	0.021 ± 0.021
	LR	**0.622 ± 0.002**	0.454 ± 0.003	0.459 ± 0.009	0.427 ± 0.007	0.496 ± 0.02
Latest, demographics	RF	0.603 ± 0.004	0.415 ± 0.005	**0.471 ± 0.016**	0.396 ± 0.007	**0.585 ± 0.049**
	TGFNN	0.586 ± 0.043	0.412 ± 0.04	0.385 ± 0.215	0.314 ± 0.176	0.499 ± 0.282
	TNET	0.594 ± 0.007	0.424 ± 0.006	0.166 ± 0.081	0.553 ± 0.069	0.105 ± 0.072
	XGB	0.587 ± 0.004	0.388 ± 0.008	0.443 ± 0.014	0.394 ± 0.004	0.508 ± 0.034
	DT	0.597 ± 0.006	0.403 ± 0.01	0.462 ± 0.014	0.41 ± 0.019	0.534 ± 0.055
	EBM	0.646 ± 0.005	0.454 ± 0.007	0.098 ± 0.117	0.296 ± 0.27	0.063 ± 0.08
	LR	0.631 ± 0.003	0.44 ± 0.004	0.477 ± 0.004	0.43 ± 0.006	0.536 ± 0.017
Phenotypes	RF	**0.651 ± 0.002**	**0.463 ± 0.006**	**0.493 ± 0.004**	**0.442 ± 0.004**	0.56 ± 0.015
	TGFNN	0.616 ± 0.005	0.421 ± 0.01	0.488 ± 0.039	0.377 ± 0.039	**0.766 ± 0.236**
	TNET	0.601 ± 0.019	0.413 ± 0.016	0.231 ± 0.184	0.442 ± 0.071	0.224 ± 0.237
	XGB	0.627 ± 0.013	0.441 ± 0.01	0.463 ± 0.014	0.425 ± 0.011	0.508 ± 0.025
	DT	0.557 ± 0.012	0.384 ± 0.012	0.366 ± 0.043	0.413 ± 0.039	0.341 ± 0.077
	EBM	**0.621 ± 0.001**	**0.453 ± 0.005**	0.138 ± 0.102	**0.647 ± 0.104**	0.085 ± 0.068
	LR	0.609 ± 0.004	0.445 ± 0.004	0.458 ± 0.005	0.415 ± 0.005	0.51 ± 0.006
Summary statistics	RF	0.615 ± 0.004	0.44 ± 0.006	**0.475 ± 0.003**	0.407 ± 0.005	0.572 ± 0.015
	TGFNN	0.601 ± 0.007	0.436 ± 0.008	0.486 ± 0.017	0.359 ± 0.035	**0.816 ± 0.227**
	TNET	0.556 ± 0.017	0.385 ± 0.011	0.147 ± 0.094	0.464 ± 0.032	0.095 ± 0.071
	XGB	0.578 ± 0.009	0.395 ± 0.011	0.436 ± 0.014	0.392 ± 0.007	0.491 ± 0.026

**Table A4:** Results from Friedman’s test on feature set mean model performance.

Metric	*p*-Value
AUROC	2.79 × 10^−20^
AUPRC	1.86 × 10^−10^
F1	3.68 × 10^−7^
Precision	1.54 × 10^−4^
Recall	5.04 × 10^−4^

**Table A5:** Statistically significant Nemenyi test results on pairwise feature set mean model performance comparison (alpha = 0.05).

Metric	Feature Set 1	Feature Set 2	*p*-Value
	Phenotypes	Summary statistics	0.001
	Phenotypes	Latest, demographics	0.001
	Summary statistics	Latest, demo., phenotypes	0.001
	Latest, demographics	Latest, demo., phenotypes	0.001
AUROC	Phenotypes	Latest, demo., statistics	0.004
	Summary statistics	Latest, demo., statistics	0.009
	Latest, demo., phenotypes	Latest, demo., statistics	0.014
	Summary statistics	All	0.001
	Latest, demographics	All	0.001
	Latest, demo., statistics	All	0.001
	Summary statistics	Latest, demo., phenotypes	0.001
	Latest, demographics	Latest, demo., phenotypes	0.006
	Latest, demo., phenotypes	Latest, demo., statistics	0.047
AUPRC	Phenotypes	All	0.005
	Summary statistics	All	0.001
	Latest, demographics	All	0.001
	Latest, demo., statistics	All	0.001
	Phenotypes	Summary statistics	0.002
	Phenotypes	Latest, demographics	0.003
F1	Summary statistics	Latest, demo., phenotypes	0.001
	Latest, demographics	Latest, demo., phenotypes	0.001
	Summary statistics	All	0.001
	Latest, demographics	All	0.001
	Latest, demo., phenotypes	Latest, demo., statistics	0.010
Precision	Summary statistics	All	0.007
	Latest, demo., statistics	All	0.001
Recall	Summary statistics	Latest, demo., phenotypes	0.004
	Latest, demographics	Latest, demo., phenotypes	0.009

**Table A6:** Results from Friedman’s test on mean model performance on all feature sets.

Metric	*p*-Value
AUROC	7.24 × 10^−27^
AUPRC	1.06 × 10^−23^
F1	5.52 × 10^−27^
Precision	4.66 × 10^−22^
Recall	5.17 × 10^−25^

**Table A7:** Statistically significant Nemenyi test results on pairwise feature set mean model performance comparison (alpha = 0.05).

Metric	Model 1	Model 2	*p*-Value
	RF	TGFNN	0.004
	LR	TGFNN	0.001
	RF	XGB	0.001
	LR	XGB	0.001
	RF	TNET	0.001
	LR	TNET	0.001
AUROC	TGFNN	EBM	0.001
	XGB	EBM	0.001
	TNET	EBM	0.001
	RF	DT	0.001
	LR	DT	0.001
	TGFNN	DT	0.045
	EBM	DT	0.001
	LR	TGFNN	0.018
	RF	XGB	0.001
	LR	XGB	0.001
	RF	TNET	0.004
	LR	TNET	0.001
	RF	EBM	0.021
AUPRC	TGFNN	EBM	0.001
	XGB	EBM	0.001
	TNET	EBM	0.001
	RF	DT	0.001
	LR	DT	0.001
	TGFNN	DT	0.018
	EBM	DT	0.001
	RF	XGB	0.001
	TGFNN	XGB	0.026
	RF	TNET	0.001
	LR	TNET	0.001
	TGFNN	TNET	0.001
	XGB	TNET	0.003
F1	RF	EBM	0.001
	LR	EBM	0.001
	TGFNN	EBM	0.001
	XGB	EBM	0.001
	RF	DT	0.002
	TGFNN	DT	0.037
	TNET	DT	0.002
	EBM	DT	0.001
	RF	TGFNN	0.008
	LR	TGFNN	0.001
	LR	XGB	0.014
	TGFNN	TNET	0.001
	XGB	TNET	0.001
	RF	EBM	0.001
Precision	LR	EBM	0.010
	TGFNN	EBM	0.001
	XGB	EBM	0.001
	RF	DT	0.037
	LR	DT	0.001
	TNET	DT	0.001
	EBM	DT	0.001
	RF	XGB	0.023
	TGFNN	XGB	0.005
	RF	TNET	0.001
	LR	TNET	0.001
	TGFNN	TNET	0.001
	XGB	TNET	0.002
Recall	RF	EBM	0.001
	LR	EBM	0.001
	TGFNN	EBM	0.001
	XGB	EBM	0.001
	TGFNN	DT	0.045
	TNET	DT	0.001
	EBM	DT	0.001
